# Modulation of Osteogenesis in MC3T3-E1 Cells by Different Frequency Electrical Stimulation

**DOI:** 10.1371/journal.pone.0154924

**Published:** 2016-05-05

**Authors:** Yu Wang, Haitao Cui, Zhenxu Wu, Naipeng Wu, Zongliang Wang, Xuesi Chen, Yen Wei, Peibiao Zhang

**Affiliations:** 1 Key Laboratory of Polymer Ecomaterials, Changchun Institute of Applied Chemistry, Chinese Academy of Sciences, Changchun, People’s Republic of China; 2 University of Chinese Academy of Sciences, Beijing, People’s Republic of China; 3 Department of Chemistry, Tsinghua University, Beijing, People’s Republic of China; Islamic Azad University-Mashhad Branch, Mashhad, ISLAMIC REPUBLIC OF IRAN

## Abstract

Electrical stimulation (ES) is therapeutic to many bone diseases, from promoting fracture regeneration to orthopedic intervention. The application of ES offers substantial therapeutic potential, while optimal ES parameters and the underlying mechanisms responsible for the positive clinical impact are poorly understood. In this study, we assembled an ES cell culture and monitoring device. Mc-3T3-E1 cells were subjected to different frequency to investigate the effect of osteogenesis. Cell proliferation, DNA synthesis, the mRNA levels of osteosis-related genes, the activity of alkaline phosphatase (ALP), and intracellular concentration of Ca^2+^ were thoroughly evaluated. We found that 100 Hz could up-regulate the mRNA levels of collagen I, collagen II and Runx2. On the contrary, ES could down-regulate the mRNA levels of osteopontin (OPN). ALP activity assay and Fast Blue RR salt stain showed that 100 Hz could accelerate cells differentiation. Compared to the control group, 100 Hz could promote cell proliferation. Furthermore, 1 Hz to 10 Hz could improve calcium deposition in the intracellular matrix. Overall, these results indicate that 100Hz ES exhibits superior potentialities in osteogenesis, which should be beneficial for the clinical applications of ES for the treatment of bone diseases.

## Introduction

Electrical stimulation (ES) is clinically beneficial in the treatment of fracture, while 5%-10% of fractures show impaired healing and require additional orthopedic intervention. Fracture healing involves a complex multistep process. Serious periosteal and soft tissue damage at the time of fracture can lead to the formation of an atrophic nonunion. In clinic, fracture patients suffering from nonunion are mainly treated with surgery and orthopedic treatment, including bone grafts[1, 2], llizarov technique[3, 4], cytokines induction [5, 6], and biophysical therapy [7]. Because fracture patients using traditional treatments need reoperation and endure the side effects of drugs, non-invasive biophysical therapy has many advantages. ES as a popular biophysical therapy is widely used in clinical treatment of bone fracture. ES in the clinical treatment of fracture has achieved good therapeutic effect [8].

As ES is successful for healing bone fracture, the exact molecular mechanisms for ES of promoting osteogenesis remain relatively unclear. Cells differentiate into osteoblasts including the following principal development periods. First of all, cell periodic cycle and cell proliferation is activated. At the same time, extracellular matrix protein (EMC) is expressed. Secondly, alkaline phosphatase (ALP), a maker of osteoblasts proliferation, is up-regulated. In the third period, maturation of bone-like tissue, and osteopontin (OPN) and osteocalcin (OC) are maximally expressed. At last, the expression of collagenase is up-regulated and apoptotic occurs. Many research work has demonstrated that ES could promote the process of cells differentiated into osteoblasts. BRIGHTON, CT et al. show that newborn rat calvarial bone cells subjected to a matrix of sine wave 60 kHz, 20mV/CM capacitively coupled electrical signals could accelerate cell proliferation when the signal is applied continuously for six hours[9]. Recently, Charles C. Clark et al. show that human calvarial osteoblasts subjected to a capacitively coupled electric field of 60 kHz, 20 mV/cm, 50% duty cycle for 2 h duration per day significantly up-regulate mRNA expression of a number of transforming growth factor (TGF)-β family genes (bone morphogenetic proteins (BMP)-2 and -4, TGF-β1, -β2 and -β3) as well as fibroblast growth factor (FGF)-2, osteocalcin (BGP) and ALP [10]. In Sook Kim et al. show that continuous treatment of rat calvarial osteoblasts with ES of 1.5 μA/cm^2^ at 3000 Hz significantly increases cell proliferation and induces the production of VEGF[11]. Hans-Peter Wiesmann et al. show that osteoblasts are sensitive to ES (saw-tooth, 100 V, 63 ms width, 16 Hz repetition rate) resulting in an enhancement of mineralization process [12]. Ardeshir Bayat demonstrate that ES waveforms could influence bone marrow mesenchymal stem cells (BMMSCs) activities, degenerate wave and capacitive coupling show higher cell proliferation compared to other types of ES [13]. These results reveal the ES could promote cell proliferation and differentiation into osteoblasts, promote mRNA levels of osteosis-related genes, and mineralisation. However, ES parameters (such as frequency, waveform, duty cycle, and amplitude) used in previous experiments are not the same. Interestingly, a wide range of ES frequency (7.5–60 kHz) have been applied in previous experiments. In previous literatures, the role of ES frequency on osteogenesis is not clearly demonstrated. Understanding the relationship between the ES frequency and osteogenisis will most likely be very important for ES clinical application. Therefore in this study, we evaluated the role of ES frequency and determined the optimal ES frequency by analyzing mRNA levels of osteosis-related genes, ALP activity, intracellular concentration of Ca^2+^, and mineralization process. Therefore, the goal of the present study is to investigate the effects of frequency that occur during the culture of MC-3T3-E1 cells subjected to ES in vitro.

## Materials and Methods

### Equipment and settings used for ES

**Fig 1:**
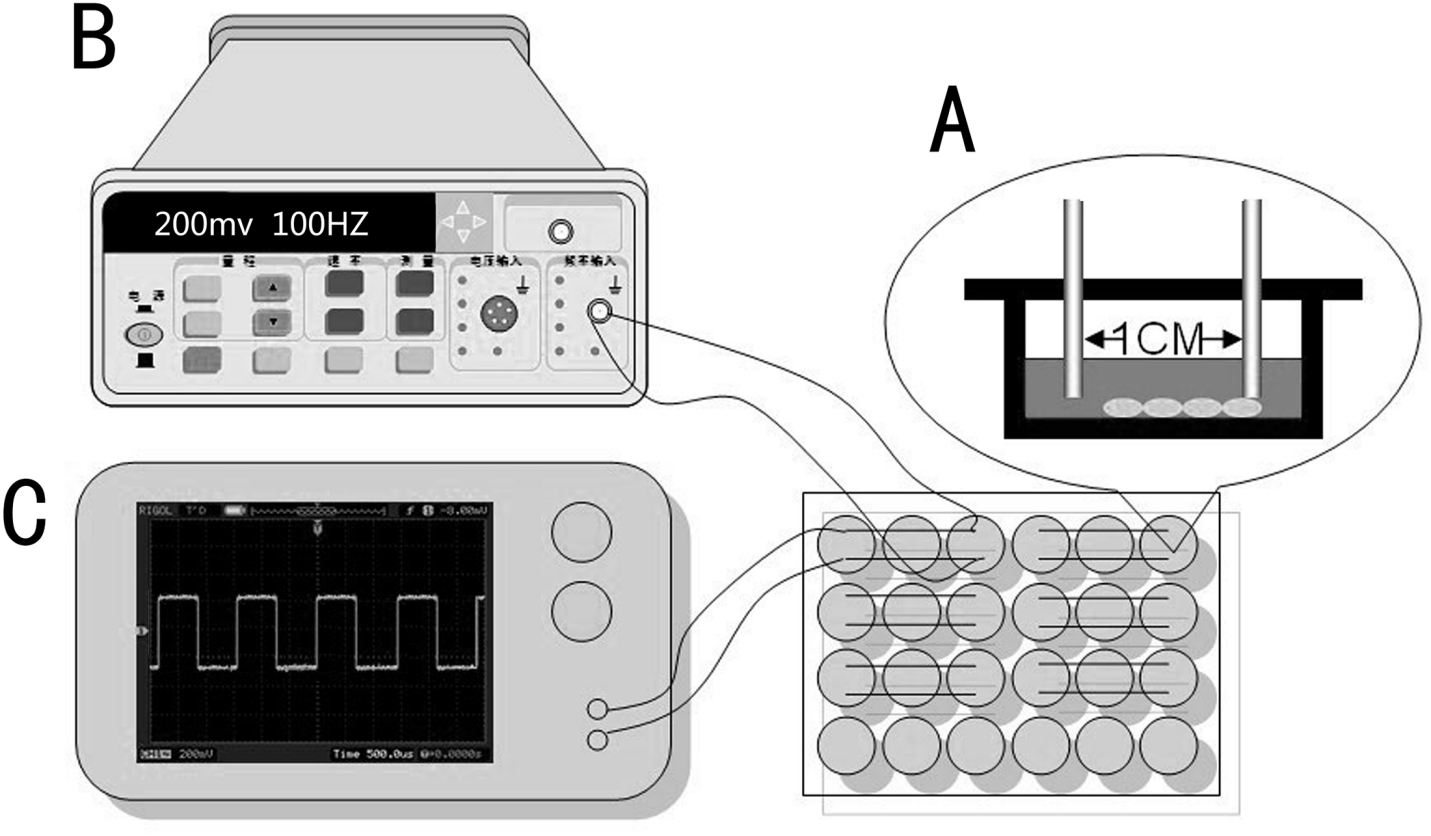
Chamber design and ES setup. (A) Platinum electrode chamber design for direct coupling; (B) ES setup including signal function generator for power supply; (C) Oscilloscope for ES verification.

The overall setup for ES is shown in Fig 1. To deliver current to a monolayer of MC-3T3-E1 cells, a pair of platinum electrodes were placed in a lid of 24 well plate and had a distance of 10 mm apart. Electrodes were directly contacted with culture medium and a uniform electric field was generated between two electrodes. For a signal source, a function signal generator (Suing, China) was connected to electrodes via alligator clips and copper wires. Output signals from generator were monitored by a digital oscilloscope (Digital Oscilloscope, Rigol, China).

### Cell culture

MC-3T3-E1 cells were obtained from the Cell Culture Centre of Institute of Basic Medical Sciences Chinese Academy of Medical Sciences (Shanghai, China). MC 3T3-E1 cells were cultured and expanded in basal medium containing Dulbecco’s modified Eagle’s medium (DMEM; Gibco) supplemented with 10% fetal bovine serum (FBS; Gibco), 100 units/ml penicillin (Sigma), and 100 mg/ml streptomycin (Sigma). Cells at passage 2–3 were grown in 24 well cell culture plate (Corning, USA) at initial seeding cell densities of 20,000 cells/well under controlled condition of temperature (37°C), a humidified atmosphere of 95% air and CO_2_ (5%). Medium was replaced with fresh medium every 2 days [14]. Cells were subjected to ES for 30 minutes per day continued 3–12 days. The ES frequency was programmed for 1 Hz to 100 kHz, and the parameters of duty cycle, amplitude, and waveform were fixed at 50%, 200 mV/cm, and rectangular pulses. When the ES parameters of duty cycle, and waveform were fixed at 50%, and rectangular pulses, amplitude (≤200 mV/cm) was optimal for investigating the role of frequency on osteogenic differentiation (S1 Fig). When the cultures in the plates reached a confluence of 65–75% (1 day after seeding), the lid of 24 well culture plates with platinum electrodes was inserted in cell plates. Cells of control group were treated exactly as above, side by side with ES group, except that the electrodes were not connected to the generator.

### Cell Proliferation Assay

Cells were subjected to different frequencies for 30 minutes per day continued 1–3 days. Next, 50μL of MTT (Sigma) stock solution in PBS (5 mg/mL) was added into each well with a final concentration of 0.5 mg/mL MTT. The cell plate was then incubated at 37°C for another 4 h. The medium was removed and 500μL of DMSO (Sigma) was added to dissolve the formazan crystals. The optical density (OD) was measured at 492 nm by a plate reader (Tecan M200). Control group was taken as negative control with 100% viability. The cell proliferation (%) of treated group was calculated by [abs] treated group/ [abs] control group×100.

### Assay of DNA synthesis

The DNA synthesis was determined by measuring the intensity of fluorescence using PicoGreen dsDNA Kit (Invitrogen). BCA Kit (Pierce) was used to measure the protein concentration of cell lysis in treated group and control group based on a BSA standard curve. The DNA synthesis can be compared by the ratio of relative fluorescence intensive and protein concentration.

### Quantification of mRNA Levels of osteosis-related genes by qRT-PCR

The mRNA of cells was extracted using trizol (Invitrogen) and collected using the Qiagen RNEasy Extraction kit (Qiagen). Samples were stored at -80°C until reversed transcription. Total RNA concentration and purity were detected by Nanodrop Assay (Tecan M200). The first strand cDNA was synthesized by reverse transcriptase as described of M-MLV manual (Promega). Gene-specific primers including GAPDH, COL I, COL II, OPN, and Runx2 were designed using the primer design software of beacon 5.0 (Table 1). All samples were performed in triplicates in 8 striped optical tube (Axygen) using qPCR SYBR Green Mix Kit (Stratagene) The amplification efficiencies of primers was verified by cDNA serial 5 times dilutions. The qPCR amplification was done as follows: initial heating at 95°C for 10min, followed by 40 cycles at 95°C for 30 s, 58°C for 60s, 72°C for 60 s. Specificity of listed oligonucleotides were checked by BLASTN^®^ (Basic Local Alignment Search Tool) against the mouse RefSeq RNA database at NCBI.

**Table 1:** List of Genes and Primer Nucleotide Sequences.

Gene Annotation	Primer Sequence (5’-3’)	Length (bp)	Reference
**COL I**	F: CGCTGGCAAGAATGGCGATC	20	NM_007742.3
	R: ATGCCTCTGTCACCTTGTTCG	21	
**COL II**	F: TGAACTAACACAGAGGAGGATCAG	24	NM_007742.3
	R: GCTTAGGGCATGAGCTTTGAC	21	
**OPN**	F: TCAGGACAACAACGGAAAGGG	21	NM_007742.3
	R: GGAACTTGCTTGACTATCGATCAC	24	
**Runx2**	F:GCCCTCATCCTTCACTCCAAG	21	NM_007742.3
	R:GGTCAGTCAGTGCCTTTCCTC	21	
**GAPDH**	F: AATGTGTCCGTCGTGGATCTG	21	NM_007742.3
	R: CAACCTGGTCCTCAGTGTAGC	21	

### Measurement of Intracellular Ca^2+^ Concentration

Intracellular Ca^2+^ concentration was evaluated on day 1 and 3 day after ES using kit (Fluo-4 NW Calcium Assay Kit, Invitrogen). Cell medium was removed, and 100 μL of the dye loading solution was quickly but carefully added to each well following the manual of kit. The plates were incubated at 37°C for 30 minutes, and then at room temperature for an added 30 min. Fluorescence was measured using plate reader with setting the exaction wavelength at 494 nm and emission wavelength at 533 nm.

### ALP Assay

MC-3T3-E1 cells were cultured in DMEM medium at an initial density of 2×10^4^ cells/well in 24-well culture plates and collected from each well after 3 days ES stimulation as described above. After the different frequency ES stimulation, the cells were assayed for ALP activity with a p-Nitrophenyl Phosphate (pNPP) Liquid Substrate System (Sigma) following the manufacturer’s instructions. First, cells were lysed in a lysis buffer (Sangon) and incubated for 30min at 37°C and centrifuged at 12,000g force for 10min at 4°C. The clear cell lysis was transferred into a new 1.5ml centrifuge tube for the following ALP assay. Add 10μl clear cell lysis into a 96-well transparent plate. Add 200 μl of pNPP solution to each well. Incubate the plate in the dark for approximately 30 minutes at room temperature. After the incubation period, read the plate at 405 nm on a multiwell plate reader (Tecan M200). The enzymatic activity was expressed as mmoles of p-NP/min/mg Protein.

### Histochemistry Assay

Histochemical analysis of ALP activity was also carried out. Cells were stained using alkaline-dye mixture of 0.01% (W/V) Naphthol AS-MX Phosphate Alkaline Solution and 0.24 mg/ml Fast Blue RR salt at 18–26°C for 30 minutes. After 30minutes, nucleuses were stained using Mayer’s Hematoxylin Solution for 10 minutes. The expression of ALP was observed by light microscopy.

## Results

### Cell proliferation and DNA content

**Fig 2:**
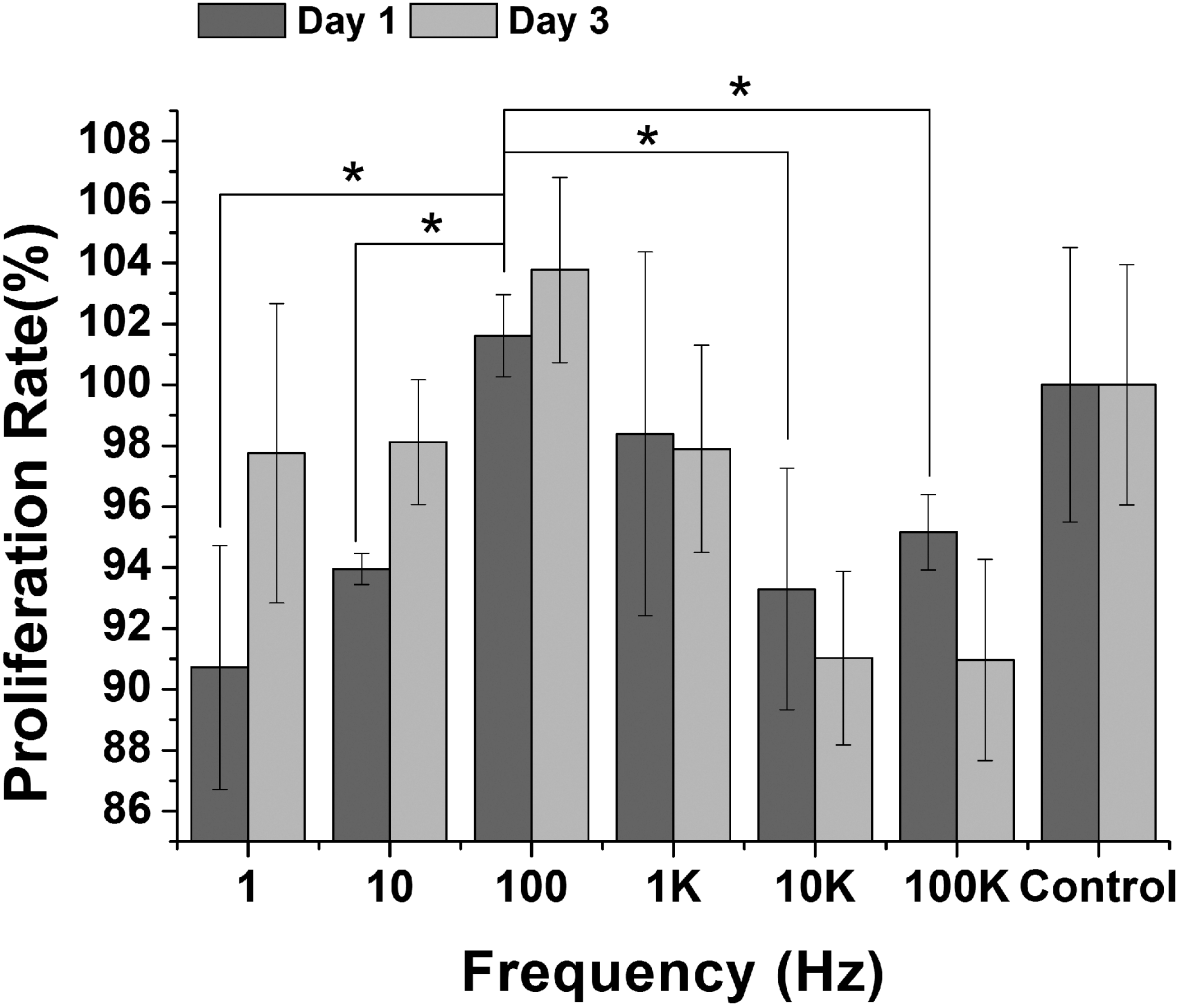
Assessment of Proliferation in MC-3T3-E1 cells after ES. MTT assay to assess proliferation of the MC-3T3-E1 cells after ES. 100 Hz group has the highest proliferation rate at days 1, 3 compared to the other frequency and control group. 1K Hz group showed similar proliferation compared to control group at days 1, 3. 1 Hz, 10 Hz, 10 KHz, and 100 KHz showed lower proliferation than control group at day 1. (P < 0.05, indicated statistically significant difference)

**Fig 3:**
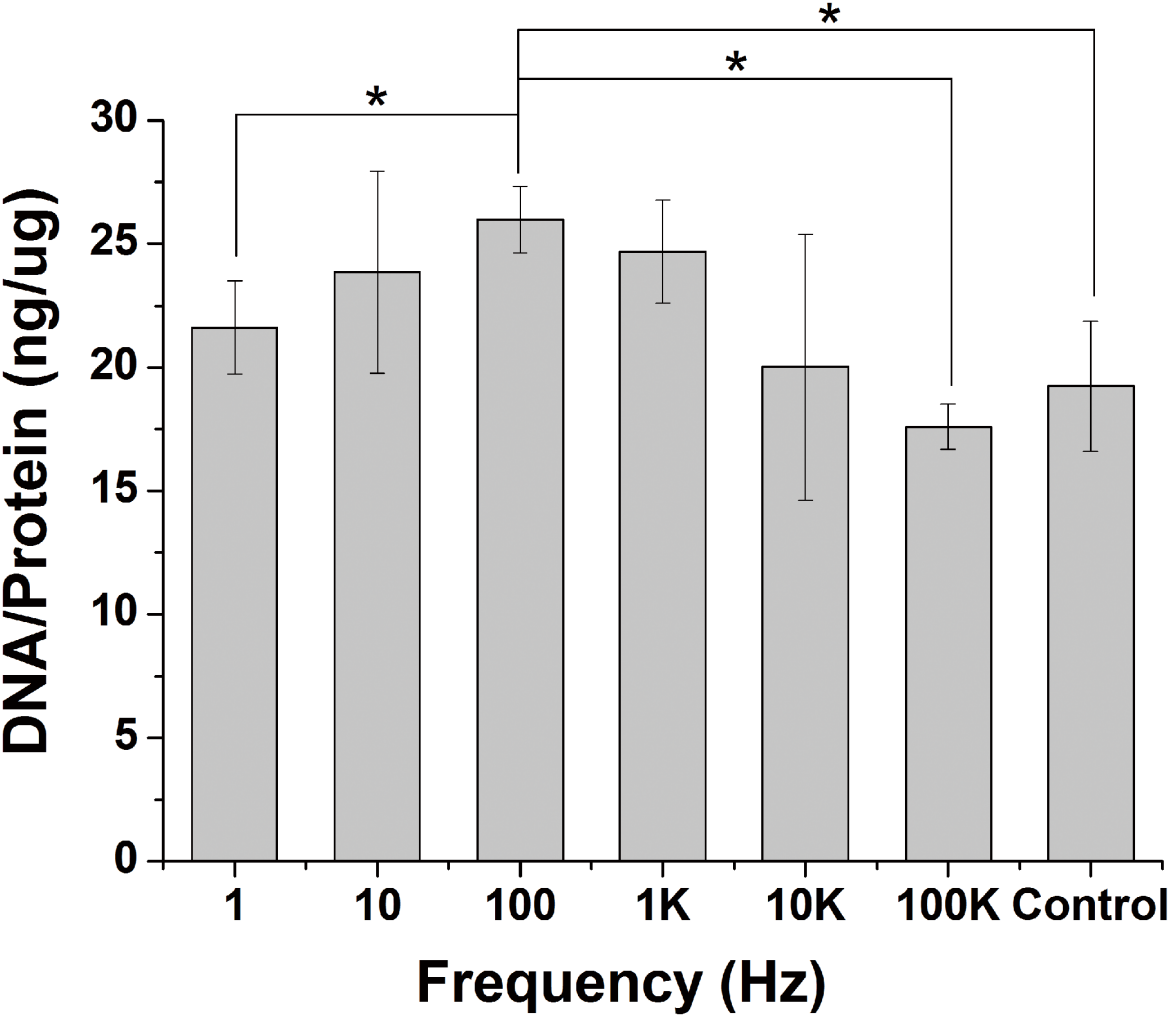
Quantitative DNA assay. Mc-3T3-E1 cells were subjected to different frequency ES for 3 days. Pico-green DNA assay was used to quantified DNA concentration of cells. 100 Hz group has the higher DNA concentration than other ES groups and control.

Cell proliferation and DNA content are important indicators in ES frequency studies. Here, we carry out a detailed analysis of cell proliferation and DNA content to determine potential effects of ES frequency. We find that cell proliferation is similar on 1 K and on control, while cell proliferation is decreased on 1, 10, 10 K, and 100 K compared to control at day 1. However, only the cell proliferation of 100Hz is higher than control group at day 1 and day 3. The cell proliferation of 100 Hz is 8.5% more than 10 Hz (P < 0.001), 12.1% more than 1 Hz (P < 0.05), 9.3% more than 10 KHz (P < 0.05), and 7.2% more than 100 KHz (P < 0.05) at day 1. Cell proliferation at day 3 has the similar tendency as day1. Cell proliferation has a maximum increase on 100 Hz compared to control. Cell proliferation is inhibited on 10 KHz, and 100 KHz compared to control at day 3 (Fig 2). We also analysis of cell viability using DNA content to determine effects of ES frequency. DNA content was determined after cells were subjected to different ES frequency (from 1 Hz to 100 KHz) for 3 days. There is no obvious change in 1 Hz, 10 Hz, 10 KHz and 100 KHz compared to control group, but 100 Hz and 1 KHz causes an increase in DNA content compared to control group. The DNA contend of 100 Hz is maximum and 1.35 fold more than control group (Fig 3).

### Quantification of gene expression

**Fig 4:**
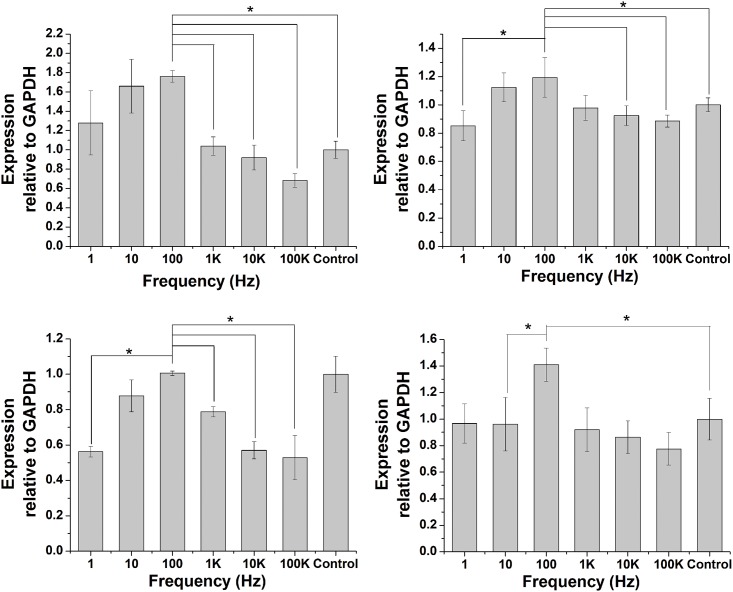
The gene expression of osteosis-related genes. MC-3T3-E1 cells were subjected to different frequency ES for 3 days. The mRNA levels of Collagen I, Collagen II, OPN, and RUNX2 were quantified by real-time PCR. The data points represent the mean value ±SD (n = 3), *P<0.05.

Collagen I, Collagen II, OPN, and Runx2 were evaluated as early osteosis-related genes (Fig 4). Collagen I was most responsive to frequency, with a maximum increase of 100Hz group in expression of 1.76-fold over control group on day 3 of culture. When frequency was fixed as 1 Hz, 10 Hz, and 100 Hz respectively, mRNA levels of collagen I were significantly higher, compared to control group. In 1 KHz and 10 KHz, mRNA levels of collagen I were not obviously different compared to control group. However, in 100 KHz treated group, mRNA levels of collagen I were slightly lower than control groups (Fig 4A).

A slightly increase in mRNA of collagen II was observed after the stimulation of 10 Hz and 100 Hz, compared to control group. The mRNA level of collagen II was not significantly regulated by the stimulation of 1 Hz, 1 KHz, 10 KHz, 100 KHz, compared to control group (Fig 4B). In 1 Hz, 10 Hz, 1 KHz, 10 KHz, and 100 KHz groups, the mRNA expression of OPN was significantly lower, compared to control group. In addition, the OPN expression of 100 Hz group was no specific regulated, compared to control group (Fig 4C). The mRNA levels of Runx2 in the 100 Hz group was significantly higher than control group. 10 KHz and 100 KHz are lower than control group. 1 Hz, 10 Hz, and 1 KHz are not different from control group (Fig 4D).

### Effect of frequency on calcium uptake

**Fig 5:**
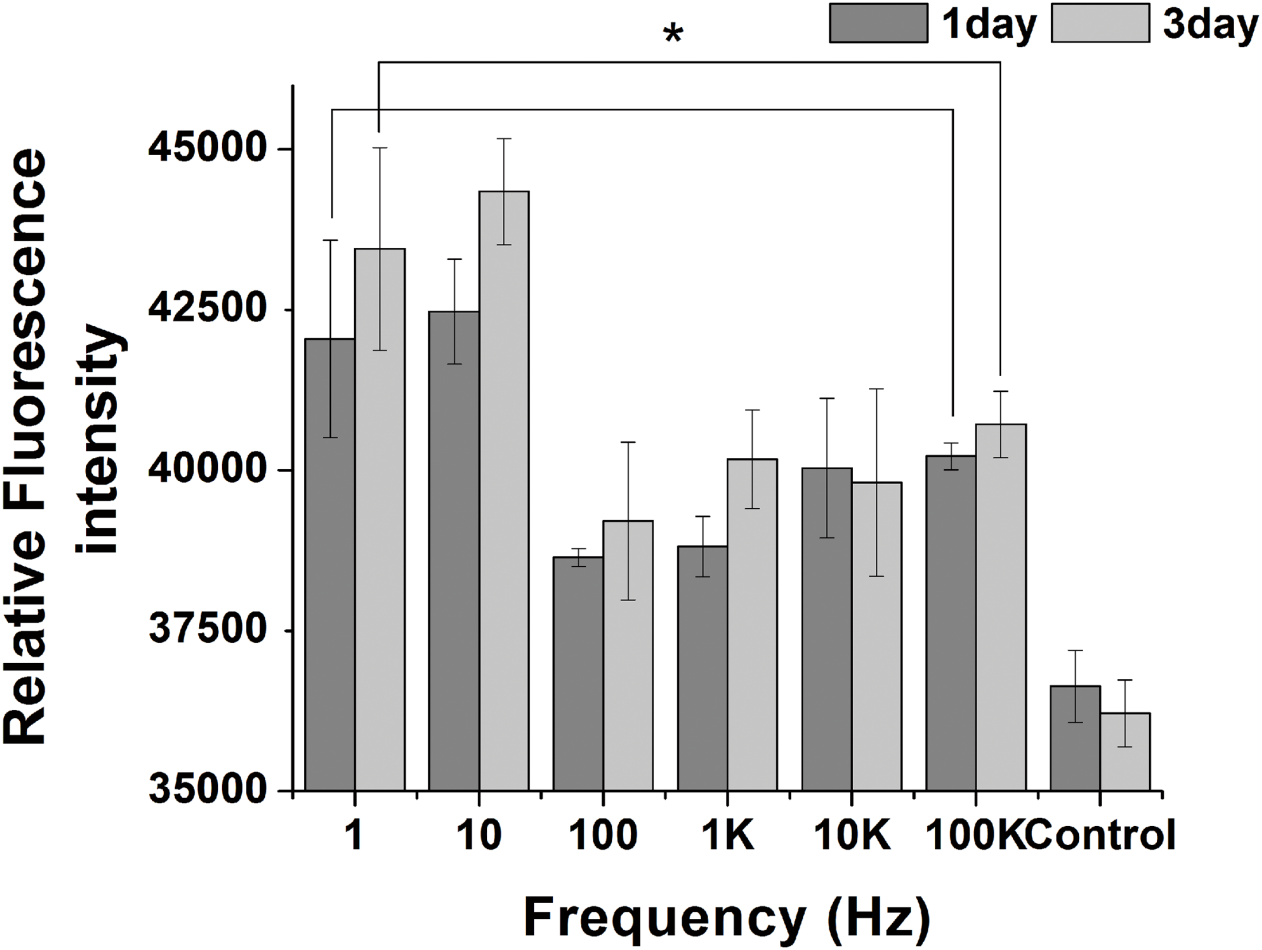
Effects of ES frequency on regulating Intracellular [Ca^2+^]. MC-3T3-E1 cells subjected to different ES frequency for 1 day and 3 days. Fluo-4 NW was used to assay relative Ca^2+^ concentration. 1 Hz and 10 Hz groups shown an increasing regulation on intracellular Ca^2+^ concentration compared to control group. * P < 0.05, indicated statistically significant difference compared to 1Hz and 10Hz.

To study the effect of ES frequency on calcium uptake by Mc-3T3-E1 cells, Fluo-4 NW calcium assay kit was used to measure the intracellular concentration of Ca^2+^. In this experiment, conditions of ES were fixed at 200mV/CM, 50% duty cycle, and rectangular waveform. The frequency of ES was variable. Mc-3T3-E1 cells were subjected to different frequency (1 Hz, 10Hz, 10 Hz, 1 KHz, 10 KHz, and 100 KHz) for 3 days. The Ca^2+^ concentration of ES groups are significantly higher than control group. The intracellular Ca^2+^ concentration of 1 Hz and 10 Hz group are greatly higher than other ES groups and are higher by 14.2% and 15.3% (P<0.05) than control group at 1 day, 19.3% and 21.8% (P<0.05) at 3 days, respectively. These results pointed out that frequency from 1Hz to 10Hz can positively up-regulate calcium uptake by MC-3T3 cells (Fig 5).

### Effect of frequency on ALP Activity

**Fig 6:**
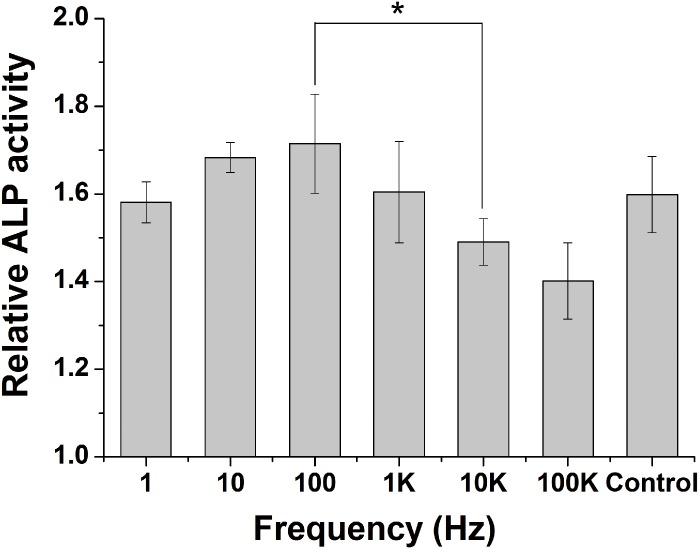
Effects of ES on ALP activity of MC3T3-E1 cells. Cells were exposed to different frequency ES for 30min a day, continued 3 days. Values are the mean ± SD of three independent cultures. *P < 0.05 vs. 1Hz, 10Hz, 100 kHz group.

**Fig 7:**
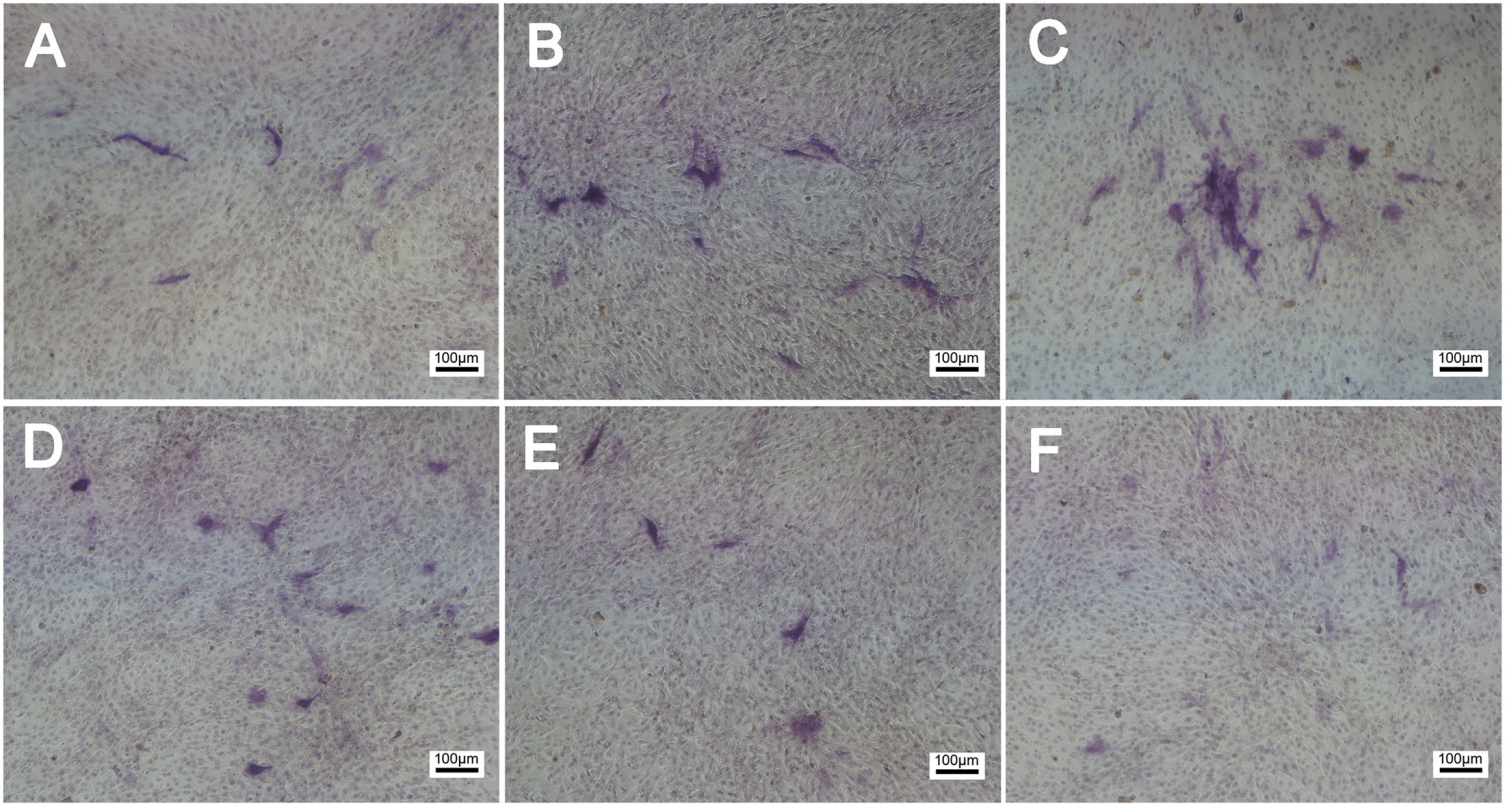
Cells were stained for ALP. ALP activity increased in a ES groups reached the highest level at 100 Hz ES for 3 days.(A)1 Hz,(B)10 Hz,(C)100 Hz,(D)1 KHz,(E)10 KHz,(F)100 KHz, bar = 100um.

The ALP activity in MC3T3-E1 cells was examined to evaluate the effect of frequency on cells osteogenic differentiation. After 3 days of ES stimulation, the ALP activity per unit protein in MC3T3-E1 cells was examined. The ALP activity of 10 Hz and 100 Hz group is increased, compared to control group. However, the ALP activity of 10 KHz, 100 KHz group is slightly decrease compared to control group. The ALP activity of 1Hz and 1 KHz does not specially differ to control group (Fig 6). Cells were histochemically stained for ALP activity to assess osteogenic differentiation. The ALP activity of ES groups are significantly increased compared to control group, with the maximal activity occurring in 100Hz group (Fig 7).

## Discussion

Previous studies show that ES play a pivotal role in regulating bone-cell’s behavior and development. However, little is known about which ES frequency is actually responsible for the bone-cell's response. This study evaluates the role of ES frequency to determine which frequency most affects osteogenesis differentiation. In our experiments, MC-3T3-E1 cells were subjected to different frequency. After ES induced, we evaluated cell proliferation, DNA content, the mRNA level of osteosis-related genes (COL I, COL II, OPN, and Runx2), markers of osteogenesis differentiation (ALP activity), and intracellular Ca^2+^ concentration.

As shown in Fig 2, if ES frequency is higher than 1 KHz or lower than 10 Hz, the signal is not stimulatory for proliferation. On the contrary, the proliferation rate obviously decrease, when cells subjected to inappropriate frequency (10 KHz or higher). Especially, when frequency is higher than 10 KHz, the proliferation is lower about 10% than control group. If the proliferation of cells was inhibited, the first step of osteogenesis differentiation would be depressed. Therefore, frequency plays an important role on proliferation of cells. 100Hz can significantly promote cell proliferation at 24h and 72h. Some previous literatures suggest that cell proliferation can promote ECM(COL-I) expression, high expression of ECM can promote the expression of ALP, high levels of ALP accelerated cells differentiated into the osteoblast, and osteoblasts mature[15–17].

From our results, frequency around 100 Hz is effective in proliferation of cells. At the same time, EMC (COL I, COL II) is also largely expressed. When frequency ranges from 10 Hz to 100 Hz, the expression of EMC (COL I, COL II) is obviously higher than control group. This promotion is also occurrence in ALP activity of Mc-3T3-E1 cells followed cultures after 3 days stimulation. Histochemical analysis of ALP activity also shows that 100 Hz can obviously enhance ALP activity. The increase of ALP activity means that osteogenesis differentiation of Mc-3T3-E1 cells is started.

Some genes play an important roles in the osteogenesis differentiation. Pluripotent mesenchymal stem cells and osteoprogenitor cells can be regulated to differentiate into osteoblasts by some key cell cytokines and functional proteins, including BMPs family, Runx2, and some ECM. In our test, ES frequency was set at a wide range from 1Hz to 100 KHz. As the results showed in Fig 4A, the frequency of 10Hz to 100Hz show an excellent capacity to induce the EMC (COL-I, COL-II) expression and osteosis-related genes (OPN, Runx2) expression. Especially of COL-I, the frequency of 1Hz to 100Hz can obviously promote COL-I expression compared to the frequency of 1 KHz to 100 KHz and controls group. Collagen is a group of naturally occurring proteins. In nature, it is found exclusively in animals, especially in the flesh and connective tissues of mammals[18]. COL I is the most abundant collagen of the human body and main component of bone. COL II makes up 50% of all cartilage protein and main component of cartilage. Elevation in COL I mRNA levels in this study proved the differentiation of MC3T3-E1 cells towards to osteogenesis differentiation.

As the results showed in Fig 4C, the expression level of OPN in the 100 Hz group is higher than other frequency, but is slightly lower than control group. Osteopontin (OPN) is a kind of non-collagenous matrix proteins. OPN expression in bone mainly occurs by osteoblasts as well as osteoclasts. OPN synthesized by osteoblasts is mainly responsible for binding hydroxyapatite. On the contrary, OPN expressed by osteoclasts plays an important role in anchoring osteoclasts to the mineral matrix of bones [19, 20]. Since OPN has the abilities of binding hydroxyapatite and anchoring osteoclasts, the high expression level of OPN will anchor large numbers osteoclasts and lead to bone resorption, the low expression level of OPN will decrease the capacity of binding hydroxyapatite. The expression level of OPN in the 100 Hz group is similar with control group, which will balance the relationship of bone resorption and osteogenesis. OPN in other frequency is obviously suppressed compared to control group, which will decrease the ability of binding hydroxyapatite.

Runx2, also called cbfa1 (core-binding factor), is an essential transcription factor for osteoblast differentiation and bone formation, which belongs to the runt-domain gene family. Runx2 expression at appropriate level, times and spaces during fetal development is essential for skeletal formation. When the expression of Runx2 was disrupted in fetal mice, the skeletal systems showed a complete lack of [21, 22]. As the results showed in Fig 4D, the mRNA level of Runx2 in the 100Hz group is higher than other frequency and control group. 100 Hz ES yields a high osteogenesis by regulating the mRNA of Runx2.

Levels of calcium ions plays an important role in the regulation of cell proliferation and differentiation of MC3T3-E1 cells [23]. Together, as a second messenger, Ca^2+^ also plays a key role in signal transduction pathways, which including of neurotransmitter release from neurons, fertilization, and contraction of all muscle cell ES. Our experiments indicate the frequency ranging from 1 Hz to 10 Hz are more capable of inducing increases in Ca^2+^. In previous literatures, the Ca^2+^ concentration has a fourfold increase exposure to electrical fields with strength of 10 V/cm, frequency of 1Hz and 10Hz to human hepatoma (Hep3B) cells for 30min compare to control. Their data suggest that the increase in Ca^2+^ induced by ac electrical fields contributes to Ca^2+^ influx from the extracellular medium, rather than release from internal stores. Considering the observation of the present study with results of our work, obviously ES with the frequency from 1Hz to 10Hz induce an increase of Ca^2+^ [24].

The molecular mechanism of ES promoting osteogenic differentiation is unclear. ES and pulsed electromagnetic field (PEMF) can alter the protein’s structure and interactions of proteins and other molecules, such as drugs, ligands and so on. We hypothesize that appropriate ES and EMF frequency can affect the cell signal transduction of osteogenic differentiation because of the structure change of osteogenic related cytokines and receptors [25]. Hong-Song Fang et al. show that PEMF (4.5-ms square pulse, repeated at 15 Hz, with a peak of 1.2 mT) can improve RUNX2 expression and suppress peroxisome proliferator-activated receptor-γ2 (PPAR-γ2) expression. PPAR-γ2 is a kind of adipogenic genes. An optimal PEMF is an effective physiotherapy in the treatment of Non-traumatic osteonecrosis of the femoral head (ONFH) by balancing the adipogenesis and osteogenesis [26]. An appropriate electrical stimulation parameter can promote BMPs binding to BMPRs, and Smad1 and Smad5 transduce BMP signals interacting with Smad4. Smads can activate transcription factor RUNX2. RUNX2 mediates the expression of osteosis-related genes. The signal transduction process is responsible for the electric stimulation promoting cells to osteogenic differentiation. On the contrary, an inappropriate electrical stimulation parameter will inhibit BMP2 mediating signaling pathway. From results of this study, 100 Hz is an optimal electrical stimulation parameter and can promote cells to osteogenic differentiation. In future study, we will verify the hypothetical molecular mechanisms of electrical stimulation promoting cells to differentiate into osteoblasts [27].

In conclusion, ES is a flexible, safety, and cheap way to treat bone fracture and nonunions by promoting cell proliferation, DNA synthesis, ALP activity, mRNA level of osteosis-genes, and the concentration of Ca^2+^. When MC3T3-E1 cells are subjected to ES in different frequency, 100Hz is better than other frequency can significantly promote the up-regulation of osteosis-related genes, activity of ALP, cell proliferation, and DNA synthesis. Our findings also indicate that 1 Hz to 10 Hz can significantly increase the concentration of Ca^2+^. Conversely, osteogenesis differentiation will be inhibited by using an inappropriate frequency of ES. From the results of our experiments, we find that frequency is the dominant factor affecting the bone-cell’s proliferation, the mRNA level of osteosis-related genes, ALP activity, and the intracellular concentration of Ca^2+^. This work approves the importance of frequency for ES to promoting osteogenesis differentiation, which is helpful for ES treatments applied to bone fracture and nonunions in clinic.

## Supporting Information

S1 Fig Assessment of Proliferation in MC-3T3-E1 cells after different amplitude ES. (TIF)
