# Supplementary figures and images for: Modulation of Osteogenesis in MC3T3-E1 Cells by Different Frequency Electrical Stimulation
Source: PLoS One. 2016 May 5;11(5):e0154924. doi: 10.1371/journal.pone.0154924 (PMC4858221; doi:10.1371/journal.pone.0154924)

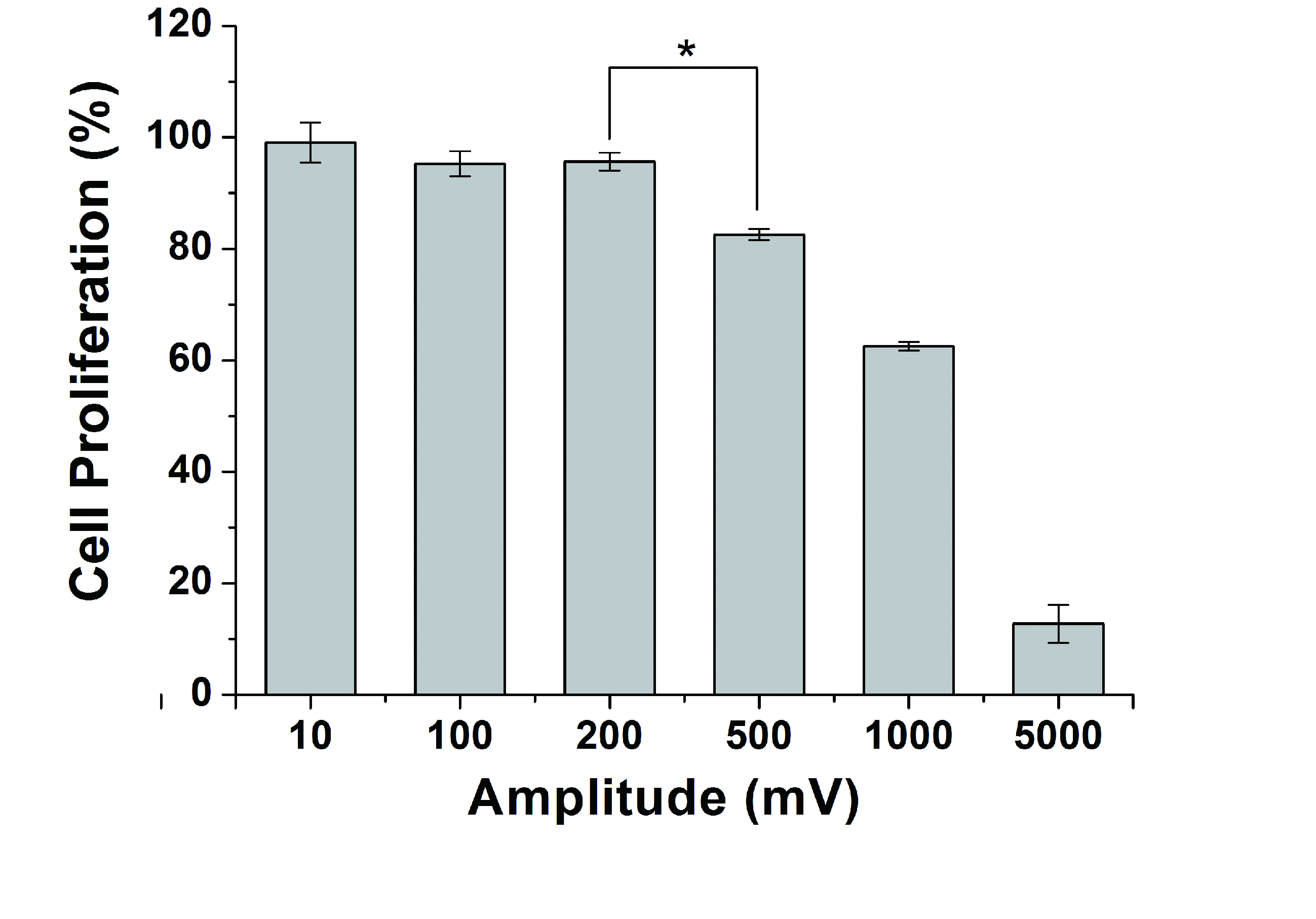

Supplement: S1 Fig — (TIF) [file pone.0154924.s001.tif]
